# Rare variants increase the risk of severe COVID-19

**DOI:** 10.7554/eLife.67860

**Published:** 2021-03-23

**Authors:** Frank L van de Veerdonk, Mihai G Netea

**Affiliations:** 1 Department of Internal Medicine, Radboudumc Center for Infectious DiseasesNijmegenNetherlands; 2 Department of Immunology and Metabolism, Life and Medical Sciences Institute, University of BonnBonnGermany

**Keywords:** TLR7, COVID-19, LASSO logistic regression analysis, Human

## Abstract

Evidence is mounting that rare loss-of-function variants in the *TLR7* gene predispose men with no medical history to severe forms of COVID-19.

**Related research article** Fallerini C, Daga S, Mantovani S, Benetti E, Picchiotti N, Francisci D, Paciosi F, Schiaroli E, Baldassarri M, Fava F, Palmieri M, Ludovisi S, Castelli F, Quiros-Roldan E, Vaghi M, Rusconi S, Siano M, Bandini M, Spiga O, Capitani K, Furini S, Mari F, GEN-COVID Multicenter Study, Renieri A, Mondelli MU, Frullanti E. 2021. Association of Toll-like receptor 7 variants with life-threatening COVID-19 disease in males: findings from a nested case-control study. *eLife* **10**:e67569. doi: 10.7554/eLife.67569

COVID-19 is a disease that does not strike equally: while most individuals will experience no or only mild respiratory symptoms, a minority – including young patients in their 20s or 30s – will develop severe pneumonia and acute respiratory distress syndrome, sometimes resulting in death. Identifying the underlying factors that predispose to severe COVID-19, especially in young individuals, is crucial to initiate preventive measures and design treatment strategies for at-risk patients.

Accumulating evidence suggests that rare loss-of-function mutations in a single gene known as *TLR7* could predispose men under 50 without known risk factors to severe COVID-19. *TLR7* codes for a Toll-like receptor that can recognise single-strand RNA present in viruses such as SARS-CoV-2, the agent responsible for COVID-19. Once activated, the receptor helps to switch on the immune response, triggering the production of pro-inflammatory molecules and the release of type I and II interferon proteins that regulate the activity of the immune system.

Now, in eLife, Alessandra Renieri and colleagues at the University of Siena and a number of other institutes in Italy – including Chiara Fallerini, Sergio Daga, and Stefania Mantovani as joint first authors – report five cases of men (three under 50, and two in their mid-60s) with severe COVID-19 who carry rare *TLR7* variants (Fallerini et al., 2021). These data validate and extend two other studies we have been involved in: one highlights two sets of two young brothers (median age of 26) carrying a rare *TLR7* variant who suffered from severe COVID-19 (van der Made et al., 2020); the other identified *TLR7* variants in a selected group of COVID-19 patients (Solanich et al., submitted to medRxiv). In total, these three reports describe eight rare *TLR7* variants in 12 male patients with no medical history who still developed severe COVID-19. We are therefore more confident with suggesting that variants of this single gene are responsible for an important proportion of risk factor for severe COVID-19 in men under 50.

Functional studies have started to shed light on the mechanism by which *TLR7* variants can lead to severe COVID-19. This work shows that the variants disrupt the production of type I and type II interferon after stimulation of the TLR7 receptor, which suggests that the mutations lead to a loss-of-function in the antiviral response to SARS-CoV-2 (van der Made et al., 2020). However, Fallerini et al. show that some of the rare *TLR7* variants only have a marginal effect on the release of type I interferon, indicating that additional pathways influence the body's defence against SARS-CoV-2.

Given these studies, it is somewhat surprising that a global initiative using exome or genome sequencing in 657 patients with severe COVID-19 did not report *TLR7* variants, focusing instead on the pathways that allow the recognition of viral infections such as influenza. This initiative identified defects in the TLR3 and IRF7 pathway that could be responsible for up to 3.5% of patients with severe COVID-19 (Zhang et al., 2020). The field is now anxiously waiting for potential *TLR7* variants to be identified in this large dataset as well. Indeed, Fallerini et al. suggest that at least 2% of severely ill Italian COVID-19 patients have loss of function *TLR7* variants, but this percentage could be even higher in other populations.

Since the first report on *TLR7* variants in severe COVID-19, diagnostic pipelines have been developed to discover such mutations. Being aware that this monogenetic factor predisposes young men to severe outcomes has several consequences. First, families of patients with *TLR7* variants could be screened, and individuals fast-tracked for vaccination if identified as carriers. Second, unvaccinated carriers of rare *TLR7* variants that predispose to severe COVID-19 could benefit from prophylactic interferon gamma treatment, similar to that given to patients with chronic granulomatous disease to prevent severe infection (The International Chronic Granulomatous Disease Cooperative Study Group, 1991; Marciano et al., 2004). Third, patients with these variants should be admitted to hospital earlier, be monitored more closely for complications, and be treated more aggressively once they become critically ill. Indeed, there is an argument for screening all men under 50 who have been admitted to intensive care (and their families) for the *TLR7* variants. Together, these measures might have a dramatic impact on clinical outcome.
